# F152. N-ACETYL-CYSTEINE SUPPLEMENTATION IMPROVES FUNCTIONAL CONNECTIVITY IN THE CINGULATE CORTEX IN EARLY PSYCHOSIS

**DOI:** 10.1093/schbul/sby017.683

**Published:** 2018-04-01

**Authors:** Emeline Mullier, Timo Roine, Alessandra Griffa, Philipp S Baumann, Paul Klauser, Philippe Conus, Kim Q Do, Patric Hagmann

**Affiliations:** 1 Lausanne University Hospital; 2 Centre Hospitalier Universitaire Vaudois (CHUV), University of Lausanne (UNIL); 3 The Dutch Connectome Lab

## Abstract

**Background:**

In schizophrenic patients, increasing evidence suggests that oxidative stress is involved on the disease pathophysiology. Estimation of the level of glutathione (GSH), main actor of the brain redox dysregulation, has revealed a decreased GSH levels in early psychosis patients (EPP). N-Acetyl-Cysteine (NAC) is an antioxidant and precursor of GSH, almost devoid of side effects. In chronic patients, add-on of NAC improved negative symptoms and reduced side effects of antipsychotics. In a recent double-blind randomized placebo-controlled trial, early psychosis patients received NAC supplementation. Whether NAC treatment leads to improvement in FC in EPP is unknown. Given the known connectivity disruptions in schizophrenia and given that GSH levels correlated with FC within the cingulate cortex, we investigate the effect of NAC supplementation during 6 months in EPP on cingulum cortex FC.

**Methods:**

20 EPP which constitutes a subgroup of the double-blind randomized placebo-controlled trial by [7] were scanned. T1-weighted volumes (1 mm in-plane, 1.2 mm slice thickness, TR/TE/TI 2300/2.98/900ms) and resting-state fMRI (gradient-echo-EPI sequence, 3.3x3.3x3.3 mm3, TR/TE 1920/30ms) recording were acquired on a Siemens-3T scanner in EPP who received either NAC (n=9) or placebo (n=11) as add-on treatment at baseline (T1) and follow-up (T2) after 6 months. A control group (n=93) was scanned in the same conditions. Data were processed with CMTK using a 68 regions parcellation according to a state-of-the-art pipeline. Time series were averaged over the Freesurfer regions and Pearson’s correlation was used as FC measure. We investigated FC changes occurring in the cingular cortices between T1 and T2 for NAC and placebo EPP and between EPP and CTRL by looking at the FC strength of these regions. Moreover, we assessed global efficiency and edge betweenness centrality (BC) for the whole brain network.

**Results:**

FC between caudal and isthmus cingulate cortices increases significantly after 6 months for patients who received NAC supplementation (p=0.01) but not for patients who received the placebo. FC of these regions was also higher in patients after 6 months of NAC supplementation (but not in placebo or baseline patients). Regarding the local network measures, BC differences are observed for the same regions. Indeed, edge BC increases between caudal and isthmus cingulate regions after NAC supplementation compared to placebo (p=0.015). At the nodal level, the increase of BC can be mainly ascribed to the isthmus cingulate cortex (p=0.01).

**Discussion:**

NAC supplementation has an impact on FC between caudal and isthmus cingulate regions in our dataset. NAC increases FC strength between these two regions. This strength is significantly higher than for matched control subjects. NAC has a stimulating effect on FC in regions along the cingulum bundle. This FC change could be partially explained by the changes in BC. The edge BC of the caudal and isthmus cingulate connection is increased for NAC subjects: a highest number of shortest paths go through this connection. It shows a growing importance of this edge in connecting the frontal and posterior regions of the brain network. A higher number of subjects would allow to confirming the robustness of these findings. This is a first study assessing the impact of NAC treatment on FC in a sample of EPP. After 6 months, NAC improves FC in caudal anterior and isthmus cingulate cortices. This FC increase is characterized by a higher BC in these brain areas, improving the connection between the frontal and posterior regions along the cingulum bundle. These results suggest that FC along the cingulum may be a biomarker for NAC treatment response.

